# Antibiotic-impregnated bone cement for preventing infection in patients receiving primary total hip and knee arthroplasty: A meta-analysis

**DOI:** 10.1097/MD.0000000000018068

**Published:** 2019-12-10

**Authors:** Jin Zhang, Xiao-Yu Zhang, Feng-Li Jiang, Yi-Ping Wu, Bei-Bei Yang, Zi-Yun Liu, Dong Liu

**Affiliations:** a Clinical Pharmacy Office, Baoji Central Hospital, Baoji; b Xi’an Jiaotong University Health Science Center; c Department of Pharmacology, Xi’an Jiaotong University Health Science Center, Xi’an, China.

**Keywords:** antibiotic-impregnated bone cement, infection, prevention, total hip arthroplasty, total knee arthroplasty

## Abstract

**Background:**

Surgical-site infections after primary total joint arthroplasty (TJA) are a significant issue. Antibiotic-impregnated bone cement (AIBC) has been widely used for the treatment of infected joints, but routine use of AIBC in primary TJA remains controversial. In this systematic review, we evaluated the efficacy of AIBC in reducing surgical-site infections after primary TJA.

**Methods:**

We systematically searched Pubmed, EMbase, Cochrane Library, CMB, CNKI, and WanFang Data for studies (published until June 1, 2019) evaluating AIBC use in reducing infection rates. Two reviewers independently screened the literature according to inclusion and exclusion criteria, extracted data, and assessed the methodological quality of included studies. Meta-analysis was performed using Review Manager 5.3 software. The registration number is CRD42017078341 in PROSPERO.

**Results:**

In total, 10 studies were included, resulting in a sample size of 13,909 arthroplasty cases. The overall pooled data demonstrated that, compared with systemic antibiotics, AIBC was more effective in decreasing deep infection rates (odds ratio [OR] = 0.35, 95% confidence interval [CI] = 0.14–0.89, *P* = .030), although there were higher superficial infection rates with AIBC (OR = 1.53, 95% CI = 1.11–2.11, *P* = .010). Compared to systemic antibiotics alone, AIBC with systemic antibiotics significantly decreased deep infection rates (OR = 0.55, 95% CI = 0.41–0.75, *P* = .0001) but there was no difference in superficial infection rates (OR = 1.43, 95% CI = 0.81–2.54, *P* = .220). In the subgroup analysis, both randomized controlled trials and cohort studies had reduced deep infection rates after primary TJA (OR = 0.61, 95% CI = 0.37–0.99, *P* = .050 and OR = 0.49, 95% CI = 0.34–0.70, *P* = .0001, respectively). AIBC decreased deep infection rates in both total hip and knee arthroplasty (OR = 0.25, 95% CI = 0.12–0.52, *P* = .0002 and OR = 0.62, 95% CI = 0.45–0.87, *P* = .005, respectively). Deep infection rates were significantly decreased by AIBC with gentamicin (OR = 0.31, 95% CI = 0.20–0.49, *P* < .00001) but unaffected by AIBC with cefuroxime (OR = 0.35, 95% CI = 0.10–1.20, *P* = .100). Deep infection rates in the AIBC and control groups were similar when laminar airflow was applied to the operating room (OR = 0.90, 95% CI = 0.60–1.35, *P* = .620); however, without laminar airflow, the efficacy of AIBC in decreasing deep infection rates was significantly higher than that of control group (OR = 0.21, 95% CI = 0.08–0.59, *P* = .003).

**Conclusions:**

AIBC may significantly decrease deep infection rates after primary total hip and knee arthroplasty, with or without systemic antibiotics.

## 1 Introduction

There are over 1.5 million cases of primary total hip and knee arthroplasty worldwide annually, and the number of cases have increased in aging populations.^[1,2]^ One serious complication after total hip and knee arthroplasty is surgical-site infection, which can result in catastrophic consequences for patients and substantial economic burden for hospitals. Surgical-site infection may correlate with prolonged hospitalization, revision surgery, reduction of the patient's functional status, and increased mortality.^[3]^ The use of antibiotic prophylaxis and improvement of operating room environments have been effective measures in reducing the incidence of surgical-site infections.^[4,5]^ However, the incidence of surgical-site infections was still estimated to be 1% to 2% among patients after total hip arthroplasty (THA) and 2% to 3% among patients after total knee arthroplasty (TKA).^[6,7]^

Antibiotic-impregnated bone cement (AIBC) leads to a locally high antibiotic concentration. In 1970, AIBC was introduced for the treatment of surgical site infection after joint arthroplasty.^[8]^ During the past 4 decades, the use of AIBC has been widely accepted in revision surgery for infections at the site of an arthroplasty.^[9]^ However, the routine use of AIBC in primary total joint arthroplasty (TJA) has remained controversial. In some European countries, the prophylactic application of AIBC in primary TJA has been standard practice for many years. However, the United States Food and Drug Administration has approved the use of AIBC in people receiving revision total hip and knee prostheses, but its use in primary TJA remains an off-label usage.^[10–12]^

The aim of this article was to determine the effect of prophylactic application of AIBC in reducing the incidence of surgical-site infection after primary TJA.

## 2 Materials and methods

### 2.1 Data sources and searches

The protocol of this review was registered in PROSPERO, with the registration number CRD42017078341 (http://www.crd.york.ac.uk/PROSPERO/). The protocol of this systematic review complied with the preferred reporting items for systematic reviews and meta-analyses statement. The electronic databases, including Pubmed, EMbase, Cochrane Library, CMB, CNKI, and WanFang Data, were searched until June 1, 2019, in English and Chinese languages. The reference lists of the included studies and the World Health Organization International Clinical Trials Registry Platform were also searched to identify potential studies. Keywords including antibiotic cement, antibiotic bone cement, antibiotic-impregnated bone cement, antibiotic-loaded bone cement, hip arthroplasty/replacement, knee arthroplasty/ replacement, joint arthroplasty/replacement, antibiotic prophylaxis, and prosthesis-related infection were used in the search. Our study was performed based on previous studies, so the ethical approval and informed consent were not required.

### 2.2 Inclusion criteria

Our inclusion criteria were as follows:

(1) randomized controlled trial (RCT) or cohort study;
(2) patients received a primary THA or TKA;
(3) bone cement used for patients;
(4) studies included a trial group that used AIBC and a control group that used bone cement without antibiotic;
(5) the outcome included the incidence of surgical-site infection.

### 2.3 Exclusion criteria

The studies were excluded if they

(1) were duplicated publications, reviews, abstracts from conferences, or animal studies,
(2) used AIBC therapeutically or in a revision total hip and knee arthroplasty, or
(3) conducted follow-up for less than 12 months.

### 2.4 Methodological quality assessment

The methodological qualities of all included RCTs were assessed using the Cochrane's tool to avoid bias assessment,^[13]^ which covered 6 specific domains including selection bias, performance bias, detection bias, attrition bias, reporting bias, and other sources of bias. All of these domains were graded as low risk of bias, high risk of bias, or unclear risk of bias.

The methodological qualities of included cohort studies were evaluated by using the Newcastle–Ottawa scale. This scale used a total of 9 stars: 4 in the method of patient selection, 2 in comparability of the study groups, and 3 in the number of outcome assessments.^[14]^

### 2.5 Statistical analysis

The statistical analyses were performed using Review Manager 5.3 software (Cochrane Collaboration, Oxford, United Kingdom). The odds ratio (OR) and 95% confidence intervals (CIs) were used to measure the outcomes. A *P*-value of less than .05 was considered statistically significant. Heterogeneity among the studies was estimated with the *I*^2^ statistic. Pooled ORs were obtained by using either a fixed-effect model (used in the absence of heterogeneity, *I*^2^ < 50%) or random-effect model (used in the presence of heterogeneity, *I*^2^ > 50%). Publication bias was measured by using an Egger funnel plot.^[15]^

## 3 Results

### 3.1 Literature searching

**Figure 1:**
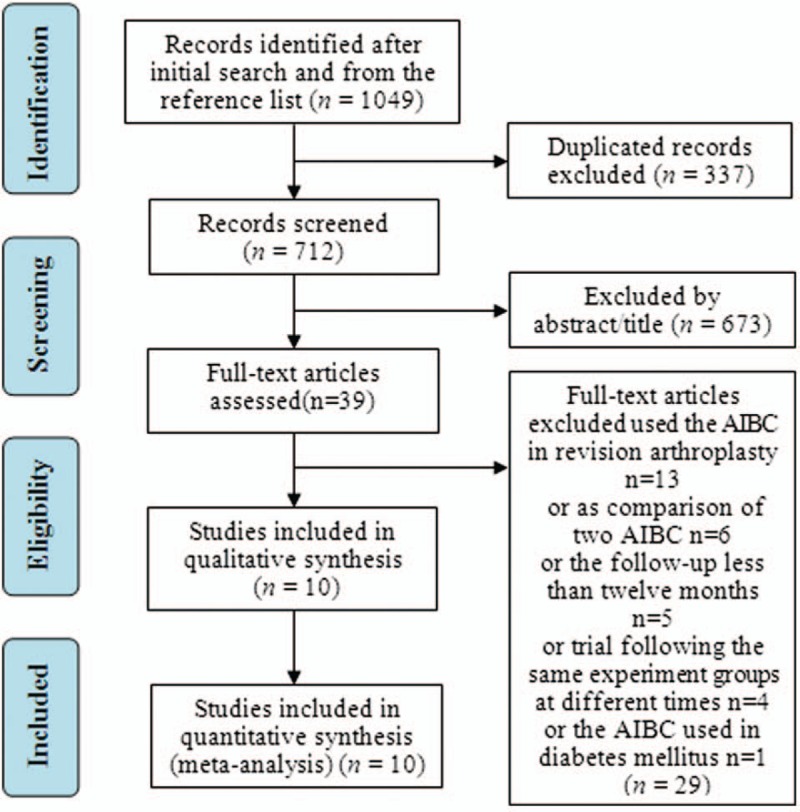
Flow diagram of the literature searching.

The literature search procedure is shown in Figure 1. A total of 1049 potentially relevant articles were identified from the aforementioned databases. After removing 337 duplicated articles, the titles and abstracts were screened from the remaining 712 articles. 673 articles were then excluded as irrelevant and 39 full-text articles were assessed for eligibility. Finally, 10 studies, meeting all the established criteria, were included in this meta-analysis.^[16–25]^

### 3.2 Study characteristics and quality assessment

In this meta-analysis with 10 included studies, 5 were RCTs and 5 were cohort studies. The major characteristics of the 10 studies are shown in Table 1. The methodological quality of the RCTs and the comparative cohort studies are shown in Tables 2 and 3, respectively.

**Table 1:**
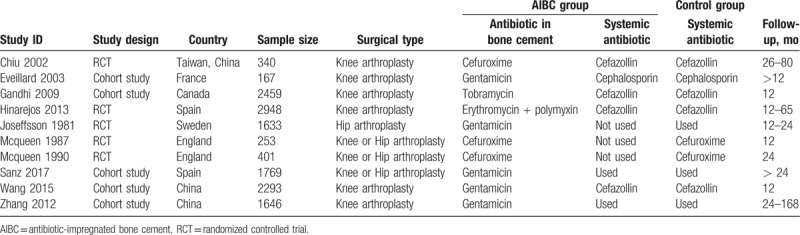
Major characteristics of the included studies.

**Table 2:**
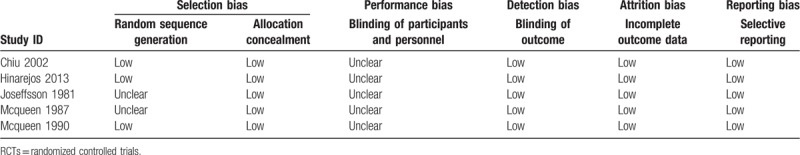
Quality assessment of the included RCTs.

**Table 3:**
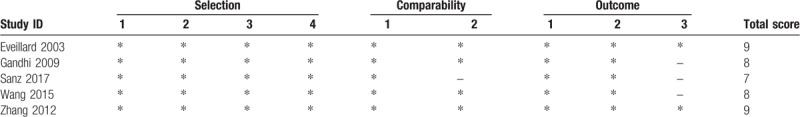
Quality assessment of the included cohort studies.

### 3.3 Superficial and deep infection rate

#### 3.3.1 AIBC versus systemic antibiotic

**Figure 2:**
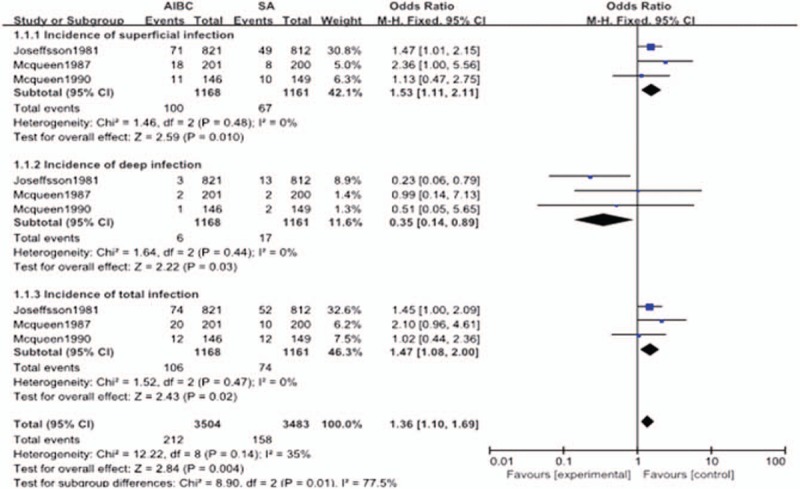
The comparison in surgical site infection between AIBC and intravenous antibiotics after joint replacement. AIBC = antibiotic-impregnated bone cement.

There were 3 RCTs included in both the superficial infection and deep infection subgroups. For the superficial infection rate, a fix-effect model was employed, in that no significant heterogeneity was observed among the subgroups (*P* = .480; *I*^2^ = 0%). The results indicated that the superficial infection rate of the AIBC group was significantly higher than that of the systemic antibiotic group (OR = 1.53, 95% CI = 1.11–2.11, *P* = .010). For deep infection, the heterogeneity between the 2 subgroups was not statistically different (*P* = .440; *I*^2^ = 0%), therefore a fix-effect model was used. The total pooled results showed that the deep infection rate of AIBC group was significantly lower than that of the systemic antibiotic group (OR = 0.35, 95% CI = 0.14–0.89, *P* = .030) (Fig. 2).

#### 3.3.2 AIBC combined systemic antibiotic versus systemic antibiotic

**Figure 3:**
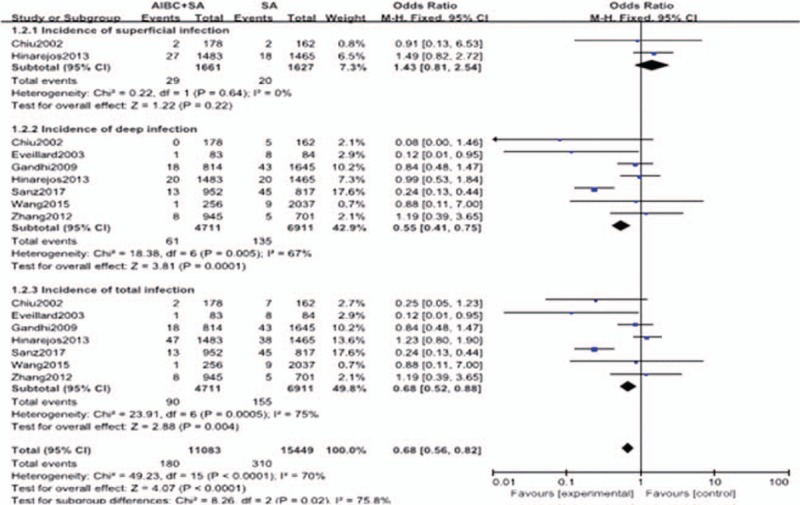
The comparison in surgical site infection between AIBC combined intravenous antibiotics and intravenous antibiotics after joint replacement. AIBC = antibiotic-impregnated bone cement.

There were 2 studies included in the superficial infection subgroup and 7 studies in the deep infection subgroup. In the superficial infection group, there was no significant heterogeneity (*P* = .640; *I*^2^ = 0%), so a fix-effect model was used. The results indicated that there were no statistical differences in superficial infection rates between the AIBC combined with systemic antibiotics group and the systemic antibiotics only group (OR = 1.43, 95% CI = 0.81–2.54, *P* = .220). For deep infection, the heterogeneity between the 2 subgroups was statistically different (*P* = .005; *I*^2^ = 67%), so a random-effect model was used. The total pooled results showed that the deep infection rate of the AIBC combined with systemic antibiotics group was significantly lower than that of the systemic antibiotics only group (OR = 0.55, 95% CI = 0.41–0.75, *P* = .0001) (Fig. 3).

### 3.4 Subgroup analysis of deep infection rate

#### 3.4.1 Subgroup analysis in study design

**Figure 4:**
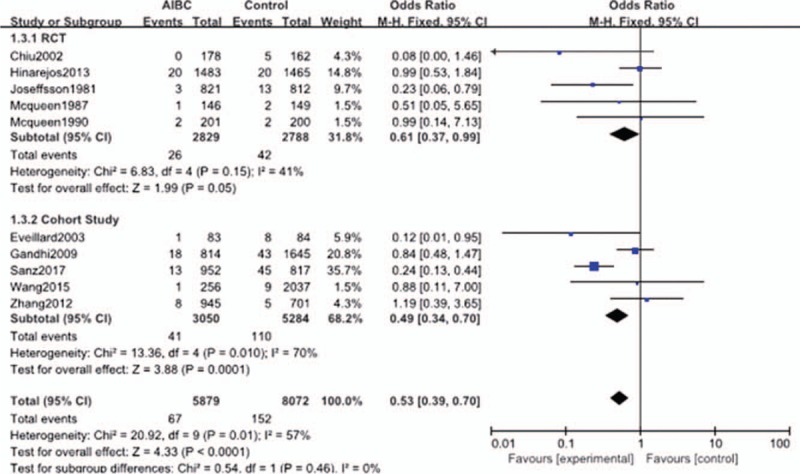
The comparison in deep surgical site infection with or without AIBC of different study designs. AIBC = antibiotic-impregnated bone cement.

There were 5 studies in the RCT subgroup. No significant heterogeneity was identified among these studies (*P* = .150, *I*^2^ = 41%); thus, a fixed-effect model was used to pool the outcomes for subgroup analysis. The results showed that there was significant difference in deep infection rates between the AIBC and control group (OR = 0.61, 95% CI = 0.37–0.99, *P* = .050). There were 5 studies in the cohort study subgroup, with significant heterogeneity (*P* = .01, *I*^2^ = 70%), so a fixed-effect model was used for subgroup analysis. The deep infection rate of the AIBC group was significantly lower than that of the control group (the group without antibiotic-impregnated) (OR = 0.49, 95% CI = 0.34–0.70, *P* = .0001) (Fig. 4).

#### 3.4.2 Subgroup analysis by surgical type

**Figure 5:**
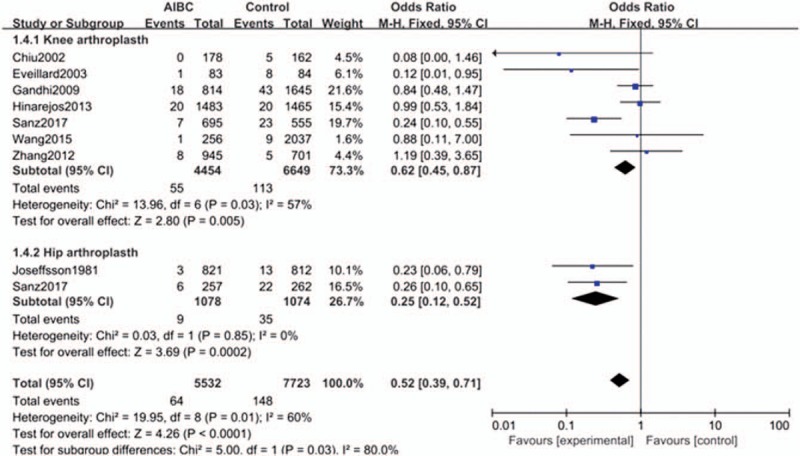
The comparison in deep surgical site infection with or without AIBC of different surgical types. AIBC = antibiotic-impregnated bone cement.

Seven studies were included in the knee arthroplasty subgroup and 2 studies were included in the hip arthroplasty subgroup. The results showed that for both knee and hip arthroplasty, the deep infection rate of the AIBC group was significantly lower than that of the control group (the group without antibiotic-impregnated) (OR = 0.67, 95% CI = 0.48–0.94, *P* = .020 and OR = 0.25, 95% CI = 0.12–0.52, *P* = .0002) (Fig. 5).

#### 3.4.3 Subgroup analysis of AIBC combined with different antibiotics

**Figure 6:**
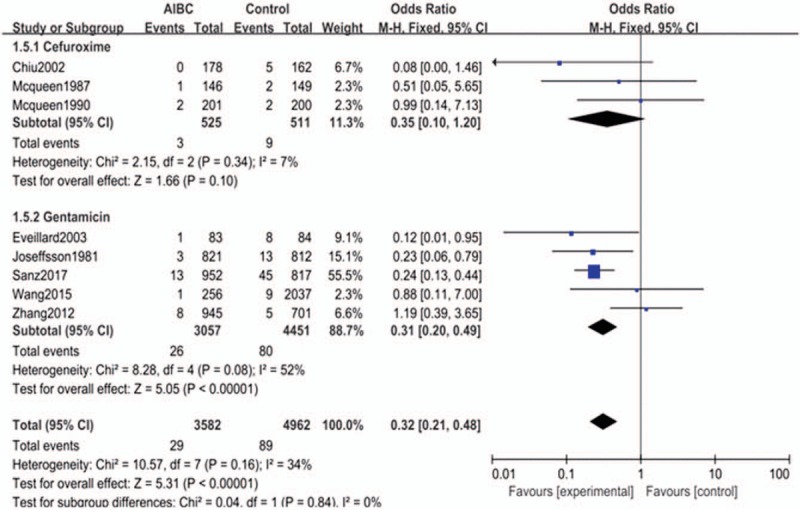
The comparison in deep surgical site infection with or without AIBC of different antibiotics. AIBC = antibiotic-impregnated bone cement.

There were 3 studies included in the cefuroxime subgroup and 5 studies in the gentamicin subgroup. Neither subgroup had significant heterogeneity (*P* = .190, *I*^2^ = 30%), thus a fix-effect model was used to pool the outcomes for subgroup analysis. The results showed that there was no statistically significant difference in the deep infection rate between the AIBC with cefuroxime group and control group (OR = 0.35, 95% CI = 0.10–1.20, *P* = .100). However, the deep infection rate of the AIBC with gentamicin group was significantly lower than that of control group (the group without antibiotic-impregnated) (OR = 0.34, 95% CI = 0.22–0.54, *P* = .00001) (Fig. 6).

#### 3.4.4 Subgroup analysis by operating room condition

**Figure 7:**
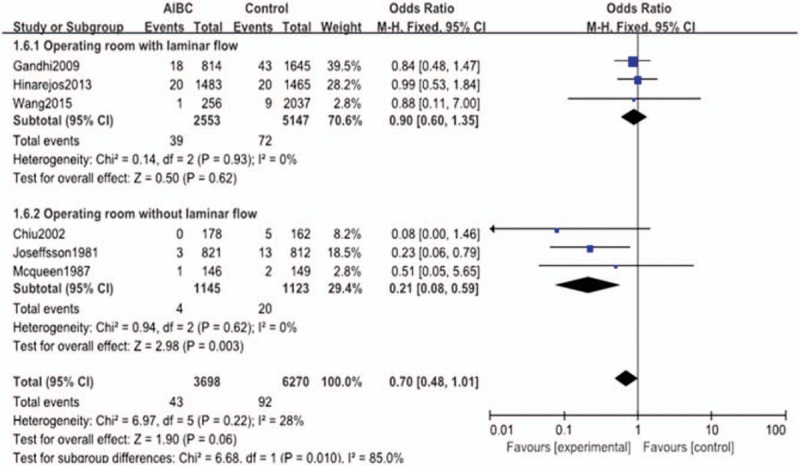
The comparison in deep surgical site infection with or without AIBC of different operating room conditions. AIBC = antibiotic-impregnated bone cement.

Three studies were included in the “operating room with laminar flow” subgroup and 3 studies were included in the “operating room without laminar flow” subgroup; neither subgroup had significant heterogeneity (*P* = .220, *I*^2^ = 28%), thus a fix-effect model was used to pool the outcomes for subgroup analysis. AIBC significantly reduced the deep infection rate in operating rooms without laminar flow (OR = 0.21, 95% CI = 0.08–0.59, *P* = .003). However, for the “operating room with laminar flow” subgroup, there was no significant effect of AIBC on the deep infection rate (OR = 0.90, 95% CI = 0.60–1.35, *P* = .620) (Fig. 7).

### 3.5 Publication bias

**Figure 8:**
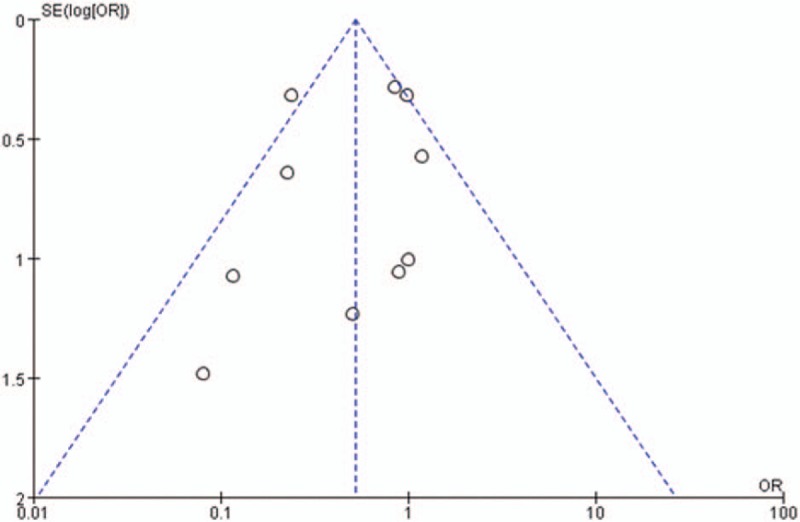
Funnel plot of studies in reducing deep surgical site infection rate with AIBC. AIBC = antibiotic-impregnated bone cement.

**Figure 9:**
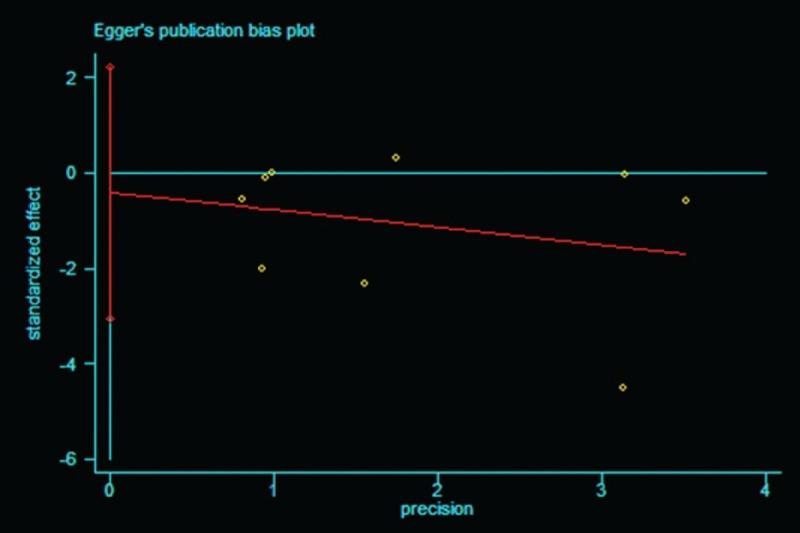
Egger test of studies in reducing deep surgical site infection rate with AIBC. AIBC = antibiotic-impregnated bone cement.

The publication bias of the included studies was evaluated by using funnel plots and Egger tests. As no asymmetry of the funnel plot was observed, the plots and the Egger test suggested that there was no publication bias in this meta-analysis (*t* = −0.307, 95% CI = −3.047 to 2.223, *P* = .722 > |*t*|) (Figs. 8 and 9).

## 4 Discussion

The effectiveness of AIBC in the treatment of joint infections has been widely accepted; however, the utility of AIBC prophylaxis in joint arthroplasty has remained controversial. Many clinical trials have begun to explore the effect of AIBC in preventing joint arthroplasty infection. However, the results of these clinical trials were inconsistent, possibly due to effects of study design, the time and district of the study implemented, the period of follow-up, different antibiotics used with AIBC, operating room conditions, or use of systemic antibiotics.^[26]^ Hence, we performed a meta-analysis to determine the value of AIBC in reducing the rate of surgical-site infection after primary TJA.

Published studies indicated that the main effect of AIBC in preventing surgical-site infections, with or without systemic antibiotics, was reducing deep infection rate. The use of systemic antibiotics could not achieve a sufficient antibiotic concentration around the bone tissue, which might be due to the inadequate blood supply of bone tissues, limiting the effects of systemic antibiotics. Since AIBC can lead to locally high concentrations of antibiotic, it may be better for reducing deep infection rates. However, compared to systemic antibiotics, AIBC was less effective in reducing superficial infection rates. The explanation could be that at areas further from AIBC, the concentrations of antibiotic were decreased, and therefore could not inhibit the growth of or kill bacteria at the superficial incision.^[26]^

Furthermore, potential factors affecting AIBC efficacy in reducing deep infection rates were revealed by subgroup analyses. Due to the absence of high-level evidence, both RCTs and cohort studies were included. Nevertheless, the results of the subgroup analysis between RCTs and cohort studies resulted in the same finding that AIBC reduced deep infection rates after TJA. In addition, from the subgroup analyses, AIBC was able to reduce deep infection rates after primary total hip and knee arthroplasty.

The effects of different antibiotics on the effect of AIBC in reducing deep infection rates were also explored via subgroup analyses. It was found that the AIBC containing gentamicin reduced deep infection rates significantly, although there was no statistical difference between cefuroxime-loaded cement and the control group. An ideal antibiotic for inclusion in bone cement should contain characteristics such as broad antibacterial spectrums, low protein binding, low sensitization potential, and high water solubility.^[27]^ Compared with other antibiotics, gentamicin contains all of these properties, as well as possessing other unique advantages such as thermal and chemical stability,^[28]^ which might be why AIBC containing gentamicin was superior in decreasing deep infection rates after primary total hip and knee arthroplasty.

For the 10 studies included in this meta-analysis, 5 studies assumed that AIBC could reduce the infection rate after primary TJA. The other 5 studies; however, deemed AIBC had no effect on decreasing the infection rate. With further investigation, we found the difference among the 10 studies was operating room conditions. The operating rooms of the 5 studies that concluded that AIBC had no effect on decreasing infection rates had laminar flow or other air cleaners. The other 5 studies with opposite conclusions had no laminar flow in the operating rooms or lacked a description of air control. We conducted subgroup analyses focused on the operating room condition: with or without laminar flow. We found that AIBC reduced deep infection rates when operating rooms lacked laminar flow but had no effect when the operating room had laminar flow. One of the possible explanations for this is that infection rates may be significantly reduced by laminar flow in the operating room, such that AIBC had no significant effect since infection rates were already reduced to a relatively low level.^[29]^

Although aiming for well-designed study, we still found some inevitable limitations in this meta-analysis. First, due to the insufficient amount of high-quality RCTs, we included both RCTs and cohort studies. The differences in study design might lead to inconsistent conclusions. Moreover, the studies included in this article spanned a large time period and different regions. Other factors, such as the antibiotics contained in AIBC, operating room condition, proficiency of operative procedure, and comorbidities of each patient, were also different, resulting in the diverse outcomes of different studies. Thus, we conducted some subgroup analyses for these factors. With an insufficient number of studies for subgroup analyses, the outcomes of subgroup analyses need to be verified further.

In addition, we found that AIBC reduced deep infection rates when laminar flow was lacking in the operating room. According to related literature reports, the main risk factors for infection after primary total hip and knee arthroplasty were body mass index above 50, tobacco use, body mass index below 20, diabetes, coronary artery disease, and operating rooms without laminar flow.^[30,31]^ Consequently, it was suggested that the effects of AIBC on infection, especially in patients with these risk factors, should be focused on in future research.

## 5 Conclusion

According to the findings described here, we believe that compared to systemic antibiotics, AIBC is less effective in preventing superficial surgical site infection but is more effective, combined with systemic antibiotics or not, in reducing deep infection rates. It appears that in operating rooms without laminar flow, effects of AIBC are more significant. In the future, larger and well-designed RCTs shall be conducted to evaluate effects of AIBC after primary TJA, especially in patients with risk factors of infection.

## Author contributions

**Conceptualization:** Dong Liu.

**Data curation:** Zi-Yun Liu.

**Formal analysis:** Jin Zhang.

**Investigation:** Zi-Yun Liu.

**Methodology:** Jin Zhang.

**Project administration:** Xiao-Yu Zhang, Feng-Li Jiang, Yi-Ping Wu, Bei-Bei Yang.

**Software:** Jin Zhang.

**Writing – original draft:** Jin Zhang.

**Writing – review and editing:** Dong Liu.
